# Mfd deficiency decreases the abundance of complete transcripts of sporulation genes and alters sporogenesis and the structure of dormant *Bacillus subtilis* spores

**DOI:** 10.3389/fmicb.2025.1680580

**Published:** 2025-10-17

**Authors:** Holly Anne Martin, Robert Heron, Hilda Leyva-Sanchez, Ryan King Perez, Amber Callaway, Tatiana Ermi, Aude Picard, Jessica Grifaldo, Jesse L. Barnes, Mario Pedraza-Reyes, Eduardo A. Robleto

**Affiliations:** ^1^Department of Physical and Life Sciences, Nevada State University, Henderson, NV, United States; ^2^School of Life Sciences, University of Nevada, Las Vegas, Las Vegas, NV, United States; ^3^Baylor College of Medicine, Houston, TX, United States; ^4^School of Medicine, University of Nevada, Reno, Reno, NV, United States; ^5^College For Health, Community, and Policy, University of Texas, San Antonio, San Antonio, TX, United States; ^6^Division of Natural and Exact Sciences, Department of Biology, University of Guanajuato, Guanajuato, Mexico

**Keywords:** Mfd, sporulation, germination, transcription-coupled repair, transcriptional regulation

## Abstract

Sporulation is a survival mechanism employed by Firmicutes, including *Bacillus subtilis*, when facing stressful conditions of growth (e.g., starvation). In this bacterium, the transcription repair coupling factor, Mfd, has been shown to play pivotal roles in sporulation transcription-coupled DNA repair and stress-associated mutagenesis. Recent studies have also revealed an unexpected role of Mfd in regulating gene expression during *B. subtilis* sporulation. This study examines the effects of *B. subtilis* Mfd deficiency on the expression of sporulation genes, sporulation efficiency, and spore morphology. In the absence of exogenous DNA damage, we found that Mfd deficiency does not compromise spore germination outgrowth; however, the loss of this factor promoted spore morphological defects and decreased sporulation efficiency. Also, our results confirmed an anomalous pattern of expression of sporulation genes in cells lacking Mfd. These results showed that Mfd influences bacterial physiology beyond DNA repair of actively transcribed genes.

## 1 Introduction

Sporulation in *B. subtilis* is a complex and highly regulated developmental process triggered by nutrient limitation, leading to a subpopulation of cells to form highly resistant structures called endospores. The process initiates similarly to cell division but diverges with the formation of an asymmetric septum, yielding a smaller forespore and a larger mother cell (Ramos-Silva et al., 2019). The forespore’s chromosome is translocated into the smaller compartment, after which the mother cell engulfs the forespore, surrounding it with a double membrane. During maturation, the forespore develops protective structures, including a thick peptidoglycan cortex, calcium-dipicolinic acid, and small acid-soluble proteins, which together confer resistance to heat, detergents, pH changes, and UV radiation (Ramírez-Guadiana et al., 2017). Once maturation is complete, the mother cell lyses, releasing the mature spore into the environment. The gene expression controlling this cell differentiation program is regulated spatially and temporally by several transcription factors that include five different sigma factors that coordinate the expression of ~400 genes to produce a dormant spore in 6–10 h (Eichenberger et al., 2004; Steil et al., 2005).

The regulation of gene expression governing the development of the spore and subsequent germination and outgrowth is controlled by factors facilitating or repressing transcription initiation (e.g., Spo0A, σᴴ, σᴷ, GerE, etc). However, we know very little about the dynamics of transcription of coding sequences of sporulation genes once initiation has occurred. The Mutation frequency decline (Mfd) factor is a transcription-coupled DNA repair (TCR) enzyme initially characterized for its role in rescuing stalled RNA polymerase (RNAP) and promoting repair of transcribed DNA strands (Selby et al., 1991; Witkin, 1994). The term “mutation frequency decline” was originally coined by Evelyn Witkin and colleagues in 1967 after observing reduced UV-induced mutagenesis in *Escherichia coli* mutants lacking this factor. A homolog of Mfd was later identified in *B. subtilis* (Ayora et al., 1996). Mfd is a double-stranded DNA-binding protein with ATP-dependent translocase activity (Deaconescu et al., 2006). It binds upstream of a stalled transcription elongation complex, translocates along the DNA, interacts with the *β* subunit of RNAP, and recruits UvrA to the lesion site (Hanawalt and Spivak, 2008; Selby et al., 2023). Mfd then facilitates RNAP release through repeated translocation cycles, clearing the way for UvrA and UvrB to initiate nucleotide excision repair (Deaconescu, 2021; Kang et al., 2021; Park et al., 2002; Selby and Sancar, 1993).

Beyond its canonical role in high-fidelity DNA repair, emerging evidence suggests that Mfd carries out other functions in the cell. Ragheb et al. (2019) demonstrated that *mfd*-deficient strains of both Gram-negative and Gram-positive bacteria required significantly more passages with subinhibitory antibiotic concentrations to develop antibiotic resistance, suggesting that Mfd promotes mutagenic processes that confer resistance. In addition to its role in promoting mutagenesis, Belitsky and Sonenshein (2011) demonstrated that Mfd influences gene expression, as its inactivation restored transcription of a gene repressed by the global regulator CodY. They proposed that Mfd collaborates with DNA-bound repressors such as CodY to remove blocked RNAP complexes, thereby terminating transcription and reinforcing repression. More importantly, this Mfd-dependent roadblock repression mechanism occurs in coding sequences and well beyond the promoter region (>+100 from the transcriptional start). Similarly, the loss of Mfd partially relieves CcpA-*cre*-dependent catabolite repression during transcription of coding sequences (Zalieckas et al., 1998). Contrarily, Mfd-deficient cells are repressed in the expression of the *leuC* (Martin et al., 2011; Pybus et al., 2010). These results suggested that Mfd mediates repression and transcription reactivation of genes at the global level. In agreement with this concept, we previously performed an RNA-Seq analysis from stationary-phase cultures (Martin et al., 2021) and showed that 1,997 genes were differentially expressed in an *mfd* knockout strain. Among these, 1,066 genes—primarily involved in protein folding, translation, and sporulation—were downregulated. Notably, 100 of the 361 known sporulation genes were affected (Hilbert and Piggot, 2004), all showing decreased expression in the absence of Mfd. Consistent with these findings, prior studies have shown that Mfd-deficient *B. subtilis* strains exhibit a ~32% reduction in sporulation efficiency compared to wild-type (Koo et al., 2017; Ramirez-Guadiana et al., 2013). Suárez et al. (2021) showed that the sporulation defect is exacerbated following disruption of the repair/prevention guanine oxidized (GO) system and main AP-endonucleases. Further, TCR of 8-OxoG lesions activate, at the onset of sporulation, a RecA-dependent checkpoint event that affected sporangia development (Suárez et al., 2021). However, the widespread downregulation of sporulation genes in Mfd mutants suggests additional regulatory roles for Mfd beyond DNA repair. Interestingly, the expression of the genes, encoding transcription factors like σE, σK, and SpoIIID, is not affected by Mfd (Martin et al., 2021) and suggests that the Mfd-dependent downregulation of gene expression is the result of altering the ability of the core RNAP to transcribe coding sequences and complete the formation of full-length transcripts.

In this study, we investigated the morphological and structural properties of spores deficient in Mfd as well as their ability to efficiently return to vegetative growth. Using a combination of sporulation and germination assays, we compared wild-type and an Mfd-deficient strain to assess differences in efficiency and spore morphology. Also, we tested gene expression patterns using RT-qPCR and visualized spore ultrastructure via transmission electron microscopy (TEM). We found that expression of genes involved in the development of the spore and the structure of the cortex was affected by Mfd. Moreover, the ability of the parent and Mfd^−^ mutant to accumulate transcripts containing the 5′ end of the transcript was not affected; however, the formation of transcripts containing the 3′ end of the transcript was decreased in the Mfd^−^ strain. This result suggests that Mfd influences transcription of genes associated with sporulation by modulating RNAP function to complete full-length transcripts. Understanding Mfd’s role in these developmental processes may offer new insights into bacterial stress responses and adaptation, with potential implications for controlling sporulation in pathogenic species.

## 2 Materials and methods

### 2.1 Bacterial strains and growth conditions

The parental strain used in this study, YB955 (Perez et al., 2024), is a prophage-cured derivative of *B. subtilis* strain 168. The *mfd* mutant strain YB9801 was derived from YB955 and carries a tetracycline (Tet) resistance cassette. The strain PERM1134 corresponds to the *mfd*-restored version of YB9801. In this strain, a functional copy of the *mfd* gene was ectopically integrated at the *amyE* locus under the control of the IPTG-inducible hyperspank (Phs) promoter. PERM1134 also carries a spectinomycin (Spc) resistance cassette for selection. The construction of the Mfd^−^ and the restored strain were described in previous reports (Ross et al., 2006). To induce *mfd* expression, isopropyl-*β*-D-thiogalactopyranoside (IPTG) was added to the growth medium at a final concentration of 0.1 mM. Strains was routinely isolated on tryptic blood agar base (TBAB), and liquid cultures were grown in Penassay broth (PAB) supplemented with 1X Ho-Le trace elements (Ross et al., 2006). When necessary, Tet (10 μg/mL) and Spc (100 μg/mL) were added to the media.

### 2.2 Sporulation assay

We measured the presence of heat-resistant spores as described previously (Suárez et al., 2021). Briefly, strains were grown in liquid Difco Sporulation Medium (DSM or NSM) at 37 °C while shaking for 24 h. Total viable CFU/mL was determined by plating serial dilutions in PBS. Cultures were then heated at 80 °C for 20 min, and viability was reassessed to quantify the number of spores in each sample.

### 2.3 Spore purification and germination assay

Strains were grown in PAB media with appropriate antibiotics and incubated overnight at 37 °C with shaking at 250 rpm. An aliquot of 300 μL was used to inoculate NSM plates and incubated at 37 °C for 5–7 days. After incubation, 5 mL of ice-cold sterile ddH_2_0 were added to the plates, and the cells were collected without agar into centrifuge tubes. Cells were spun down at 8000 rpm for 5 min, and the pellet was washed 2× in ice-cold sterile ddH_2_0. The washed cells were resuspended in 5 mL of 20% Histodenz (Sigma) solution, which was dispensed on top of a 10 mL 50% Histodenz solution. The gradient was spun down for 35 min at 11500 rpm. The supernatant was discarded, and the pellet was washed in 50 mL of ddH_2_O at least 5 times. Spores were kept in ddH_2_O at 4 °C.

For germination assays, spores in H_2_O were heat shocked for 30 min at 70 °C, cooled on ice, and inoculated into 2X Schaeffer’s glucose liquid medium supplemented with 10 mM L-alanine at 37 °C to obtain an initial OD_600_ of ~0.5. The OD_600_ of cultures was monitored with a plate reader (Synergy HTX plate reader) over 3.5 h and the values were plotted as a fraction of the initial OD_600_ (OD_600_ at time t/initial OD_600_) versus time (Suárez et al., 2021).

### 2.4 Spore preparation, TEM imaging, and analysis

A pre-inoculum of 3 mL of the parent and Mfd^−^ in PAB containing Tet (10 μg/mL) was grown overnight at 37 °C shaking at 250 rpm. Then, 600 μL of the overnight culture was added to 4 mL of Nutrient Sporulation Medium (NSM) and incubated for 24 h at 37 °C shaking at 250 rpm. Cultures (4 mL) were centrifuged at 4,000 × g for 10 min, and the resulting pellets were resuspended in a fixative solution containing 4% glutaraldehyde in 0.1 M cacodylate buffer (pH 7.4; Electron Microscopy Sciences). Cells were incubated in the fixative overnight at 4 °C, then centrifuged at 4,000 × g for 5 min, rinsed with 0.1 M cacodylate buffer, and subjected to secondary fixation in 2% osmium tetroxide (OsO₄) in 0.1 M cacodylate buffer for 1 h.

Following fixation, cells were rinsed and stained with uranyless (a lanthanide-based alternative to uranyl acetate) for 1 h. After additional rinses, samples were dehydrated through a graded ethanol series (50, 70, 95, 100%), followed by exchange into 100% acetone. Samples were then infiltrated with resin-acetone mixtures before being embedded in pure resin (Embed 812, Electron Microscopy Sciences) and cured at 60 °C for 48 h. Thin sections (70 nm) were prepared using a Leica EM UC7 ultramicrotome and imaged on a TEM JEM 1400 Plus operating at 120 keV at the Electron Microscopy Core Laboratory at the University of Utah.

To assess spore morphology, measurements of spore diameter, radius, and cortex thickness were conducted using ImageJ software. Prior to analysis, the scale was calibrated using the scale bar provided in each micrograph to ensure accurate measurements were taken. Thirty-two individual spores from both the wild-type and the Mfd*^−^* strains were manually measured using the line tool along the smallest diameter. Each measurement was performed on clearly defined spores, and values were recorded for comparative analysis between the two strains.

### 2.5 Read distribution and descriptive statistics

We focused on *B. subtilis* genes in the regulons affected by Spo0A, σ^H^, σ^E^, σ^F^, σ^K^, and σ^G^ as reported by Elfmann et al. (2025) and cross-referenced them with genes whose transcription is affected by Mfd as we reported previously (Martin et al., 2021). This process yielded 503 genes (see Table S1 Supplementary material). From our previous RNA-seq data set (Martin et al., 2021), we used the dataset to examine variability of transcription across the gene’s coding region, allowing for fine-resolution assessment of expression patterns. A custom Python script was developed to preprocess these profiles by cleaning and standardizing the data format, producing one CSV file per gene with aligned read coverage values across six biological samples (three from the wild-type strain YB955 and three from the Mfd^−^ mutant). These processed files were then used as input for a downstream analysis pipeline, which calculated the coefficient of variation (CV) (defined as the standard deviation divided by the mean) and the range (defined as the difference between the maximum and minimum read values observed across replicates for each gene).

The resulting data was visualized using Plotly to generate interactive scatter plots, with each gene represented as a point positioned by its calculated CV (*x*-axis) and range (*y*-axis) values. Genes were color-coded based on whether they met predefined variability thresholds (CV ≥0.11 and range ≥2,000) in the wild-type strain (YB955), the Mfd^−^ mutant, both, or neither. For the range threshold, we selected a value of 2,000, which represents 39% variation around the median number of reads for all the selected genes, 5,147. We also selected 11% as the threshold for the CV value as values higher than 10% indicate large variability around the mean (see Supplementary Image 1).

### 2.6 RT-qPCR assay

Transcript levels were measured using the qScript One-Step RT-qPCR Kit and following the manufacturer’s protocol (Quantabio). A total of 2 mL of T_90_ cultures grown on Penassay Broth was harvested (T_90_ indicates 90 min after the cessation of growth), and the cell pellets were resuspended in RNAlater (Thermofisher). We used Penassay broth to match the conditions used in our previous RNA-seq report (Martin et al., 2021). Total RNA was extracted using MP FastRNA Pro Blue Kit and treated with RNase inhibitor and DNase to remove contaminant DNA (Waltham, MA). RT-qPCR was performed using the Bio-Rad CFX96 Real-Time System machine, and the data were collected using Bio-Rad CFX Manager Version 3.1.1517.0823. Relative gene expression was quantified using the 2^−ΔΔCT^ method (Wacker and Godard, 2005). Transcript levels were normalized to *rnpB*, which served as an internal control. Three biological and three technical replicates were performed for each condition. Primers were designed to amplify transcripts as follows: *asnO* A1 (+163 to +332 bp) and A2 (+1,069 to +1,209 bp); *cotT* A1 (+41 to +103 bp) and A2 (+176 to +247 bp). Primer efficiencies were tested and confirmed to be similar across both primer sets, validating their use for quantitative comparisons. PCR amplification efficiency for each primer pair was assessed using a standard curve method (Livak and Schmittgen, 2001), ensuring that the target gene primers had similar efficiency to those used for the internal control gene. The primers used in these assays are described in Supplementary Table S4.

### 2.7 Statistical analysis

The significance cutoff for the Student’s *t*-test and one-way ANOVA with Tukey *post-hoc* was set at *p* ≤ 0.05. All statistical analyses were performed using GraphPad Prism 10 (Version 10.2.1; Boston, MA).

### 2.8 Identification of non-B DNA motifs

We used the web-based tools QGRS Mapper (Kikin et al., 2006) and nBMST (Cer et al., 2012) to identify potential non-B DNA motifs within the nucleotide sequences of the genes of interest. Predicted motifs were analyzed with the ViennaRNA Package 2.0 (Lorenz et al., 2011) to calculate minimum free energy (MFE) structures. Motifs below −5 kcal/mol indicate stable, physiologically relevant secondary structures (Cer et al., 2012; Du et al., 2013; Jeanneau et al., 2022; Lorenz et al., 2011).

## 3 Results

### 3.1 The effect of Mfd on sporulation

**Figure 1:**
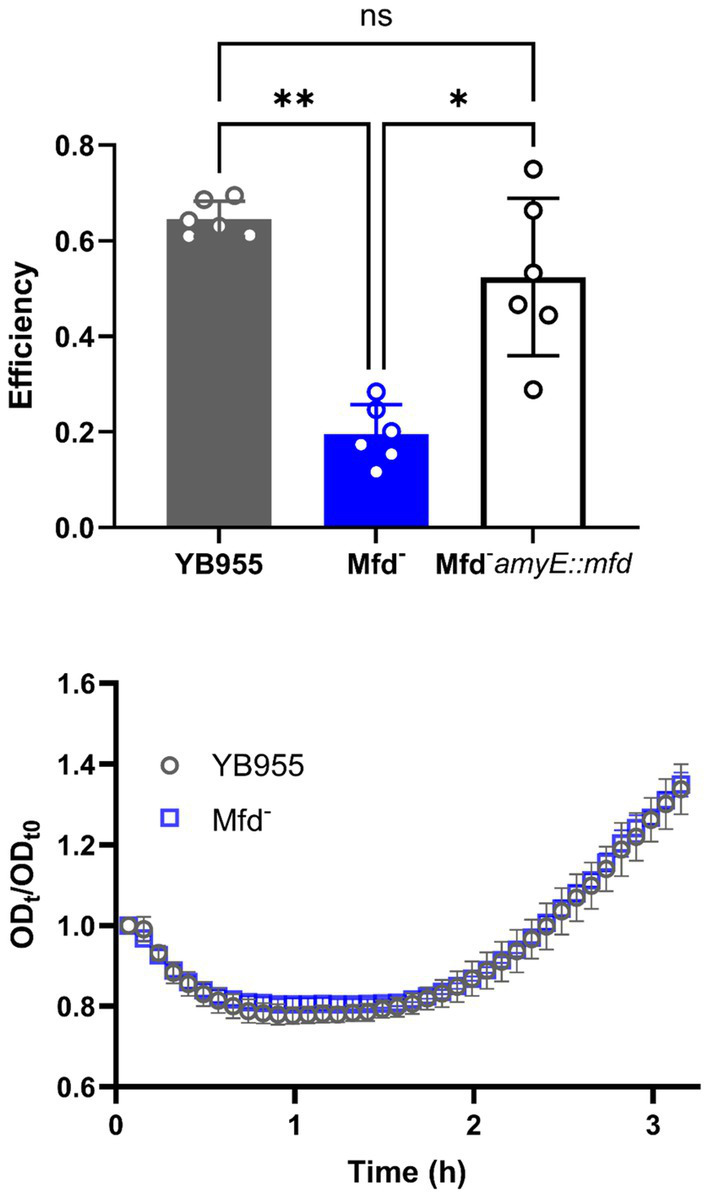
(Top) The sporulation efficiency of YB955 (Wt), YB955 *mfd:tet* (Mfd*^−^*), and YB955 *Mfd-amyE:*pHS *mfd*. Restored strain was induced with 0.01 mM IPTG. The chart shows results from six biological repetitions and error bars show standard deviation. Statistical analysis was performed using one-way ANOVA with Tukey *post-hoc*; *, ** indicate significant differences between means at *p* ≤ 0.05 and *p* ≤ 0.01, respectively. (Bottom) Germination assay measurements of YB955 and *mfd*-deficient spores. Optical density of purified spores was measured over 3 h in 2X Schaeffer’s glucose media. Data points are the averages of three biological replicates. Error bars denote standard deviation (SD).

To confirm the reported sporulation deficiency in an *mfd*-mutant *B. subtilis* YB955 strain, a sporulation efficiency assay was conducted. Briefly, this assay estimates the viable cell count of the strains analyzed before and after a pulse of wet heat that kills vegetative and non-sporulated cells (Valenzuela-García et al., 2018). A significantly lower percentage of the bacterial population produced heat-resistant spores in the Mfd-deficient strain than the wild-type, as previously reported (Figure 1, top). We also observed a return to the wild-type strain phenotype when we restored *mfd* in a single copy into the chromosome.

To determine whether the *mfd*-deficient strain produced fewer spores and to rule out any defects in germination, we first purified spores from both wild-type and the *mfd* mutant strains. Spore preparations were separated from vegetative cells using a Histodenz^®^ density gradient, ensuring that only mature spores were included in the germination assay. This step was critical, as the assay relies on measuring changes in optical density (OD₆₀₀) to detect spore rehydration and the onset of outgrowth (Valenzuela-García et al., 2018). Following purification, germination assays were performed and revealed no significant differences in germination or outgrowth rates between the wild-type and *mfd* mutant strains (Figure 1, bottom).

### 3.2 The effect of Mfd on spore morphology

**Figure 2:**
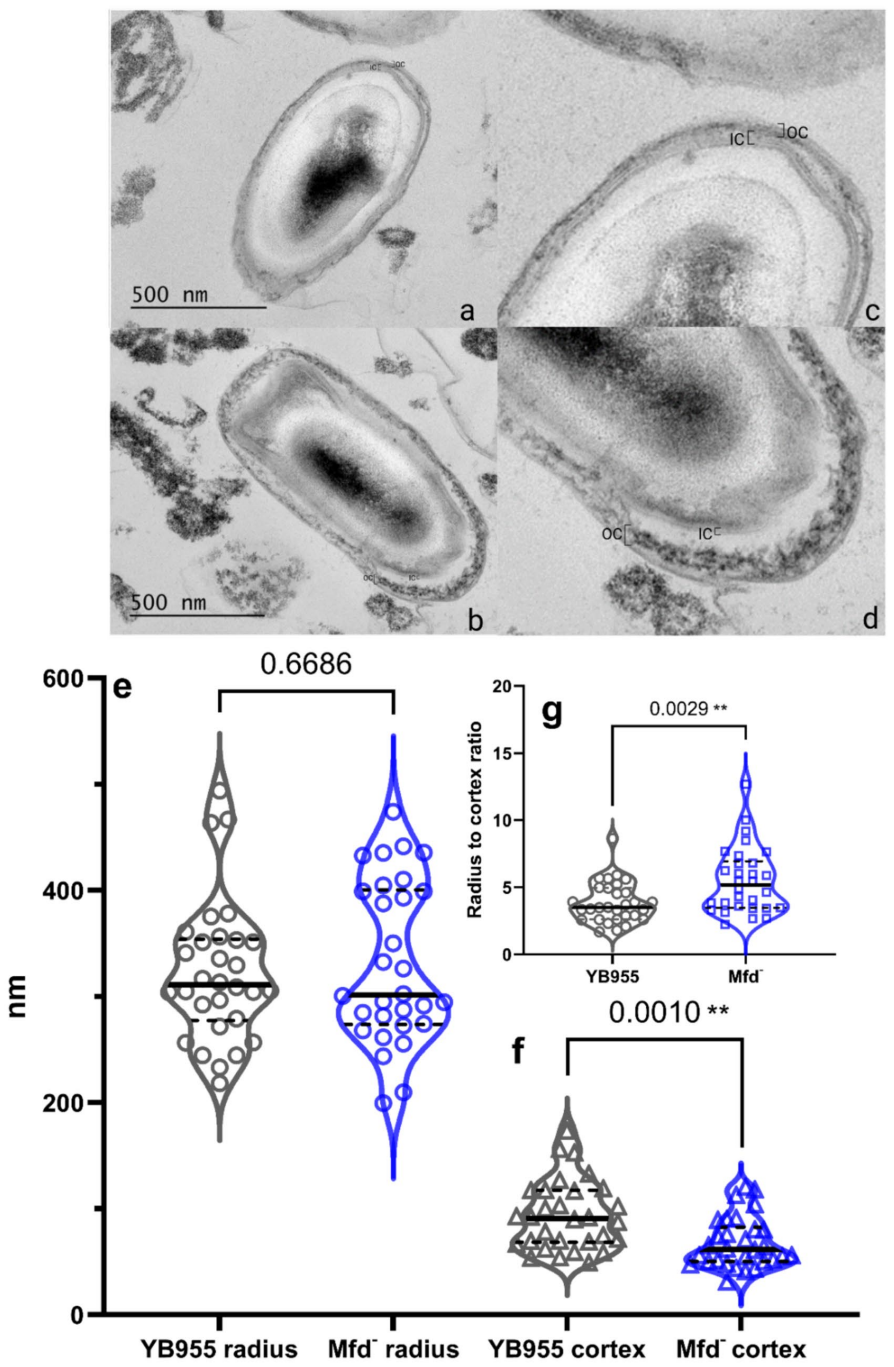
(Top) Representative TEM image of YB955 **(a,c)** and Mfd^−^ **(b,d)** *B. subtilis* spores. *B. subtilis* cultures were grown in NSM for 24 h at 37 °C, fixed in 4% glutaraldehyde and 2% OsO₄, stained with uranyless, and embedded in resin. Thin sections (70 nm) were imaged by TEM (JEM 1400 Plus, 120 keV). Brackets indicate the inner and outer cortex. (Bottom) Measurements of radii, cortex, and radius-cortex ratio from wild-type and Mfd^−^ spores from TEM images. Spore morphology as described by radius and cortex thickness were measured in ImageJ from calibrated micrographs (*n* = 30 spores per strain). Radius **(e)**, cortex **(f)**, and radius to cortex ratio **(g)**, were compared using a *t*-test. Asterisks indicate significant differences at *p* ≤ 0.01.

To identify potential morphological differences between *B. subtilis* wild-type and Mfd^−^ spores, we prepared spore samples free of vegetative cells from both strains as indicated in Materials and Methods. Then, both spore preparations were subjected to transmission electron microscopy (TEM) analyses. Visual analysis suggested that the Mfd^−^ spores displayed less uniformity in inner and outer coat layers (Figure 2, top; see brackets). We followed our visual analysis with measurements of spore radii and cortex thickness using ImageJ. For example, the median for the radius of spores (center of the spore to the nearest edge) were 310 nm and 301 nm for the parent and Mfd^−^ mutant, respectively; these were not significantly different. Contrastingly, the median cortex measurements (brackets in the TEM images) were 91 nm for the parent and 61 nm for the Mfd^−^ mutant; these values were significantly different. These differences in the cortex translated into thinner spores as shown by radii to cortex ratio; Mfd^−^ spores displayed the same radii but thinner cortex, suggesting cortex underdevelopment (Figure 2, bottom).

### 3.3 The effect of Mfd on the expression of sporulation-associated genes

**Figure 3:**
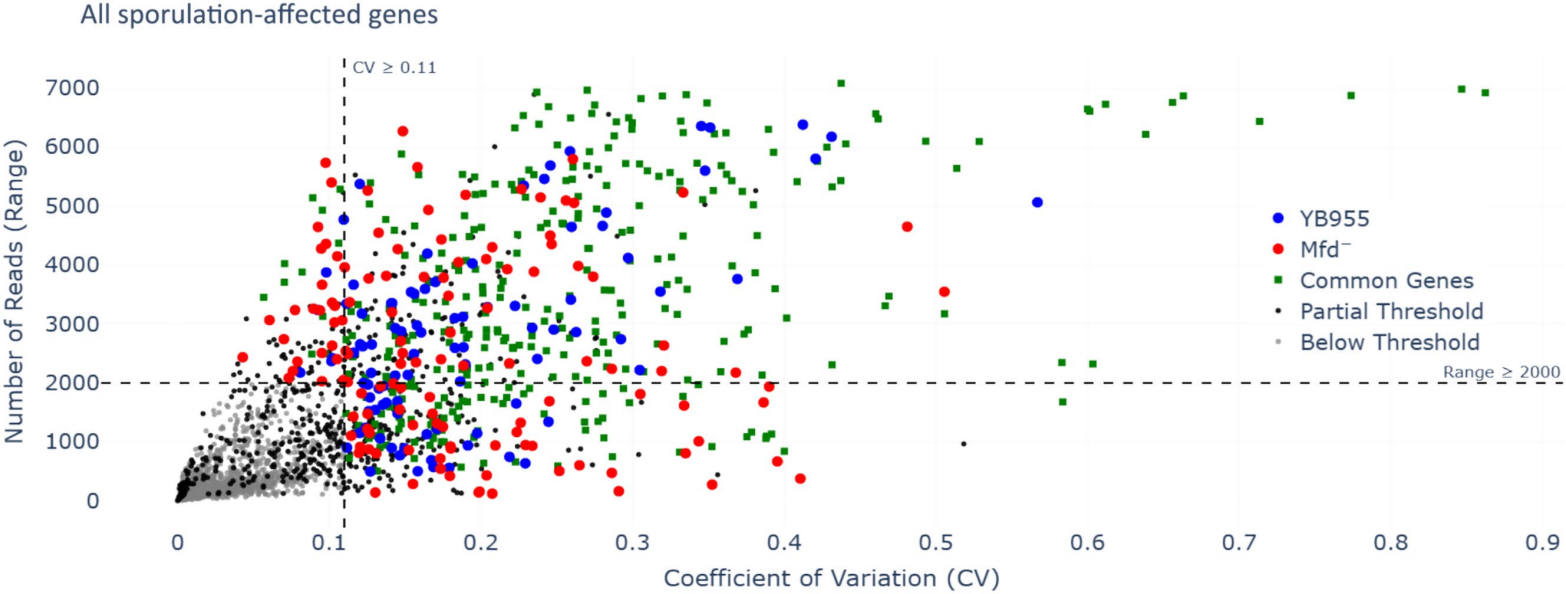
Range of RNA-seq reads in each gene and their coefficient of variation as a function of Mfd. Each dot represents one of three biological replicates measuring expression by RNA-seq of 504 genes; these comprised of the overlap of the genes contained in Spo0A, σ^H^, σ^E^, σ^F^, σ^K^, and σ^G^ regulons (Elfmann et al., 2025) and those affected by Mfd (Martin et al., 2021). The RNA-seq and its conditions are described by Martin et al. (2021). We used the unadjusted reads and considered genes showing a range of ≥2000 or a CV of ≥0.11 to display altered dynamics of transcription specific to coding sequences (beyond the promoter regions). Gray dots indicate that each of the three replicates for the same gene did not meet either the range or CV threshold—genes with low variation in RNA-seq reads (below threshold). Black dots indicate that at least one of three replicates for the same gene met one or both thresholds (partial threshold). Green dots indicate that the three replicates met one or both thresholds for the same gene in YB955 and the Mfd^−^ strains (common genes). Blue dots indicate that the three replicates met one or both thresholds for the same gene in YB955 only. Red dots indicate that the three replicates met one or both thresholds for the same gene in the Mfd^−^ strain only.

**Figure 4:**
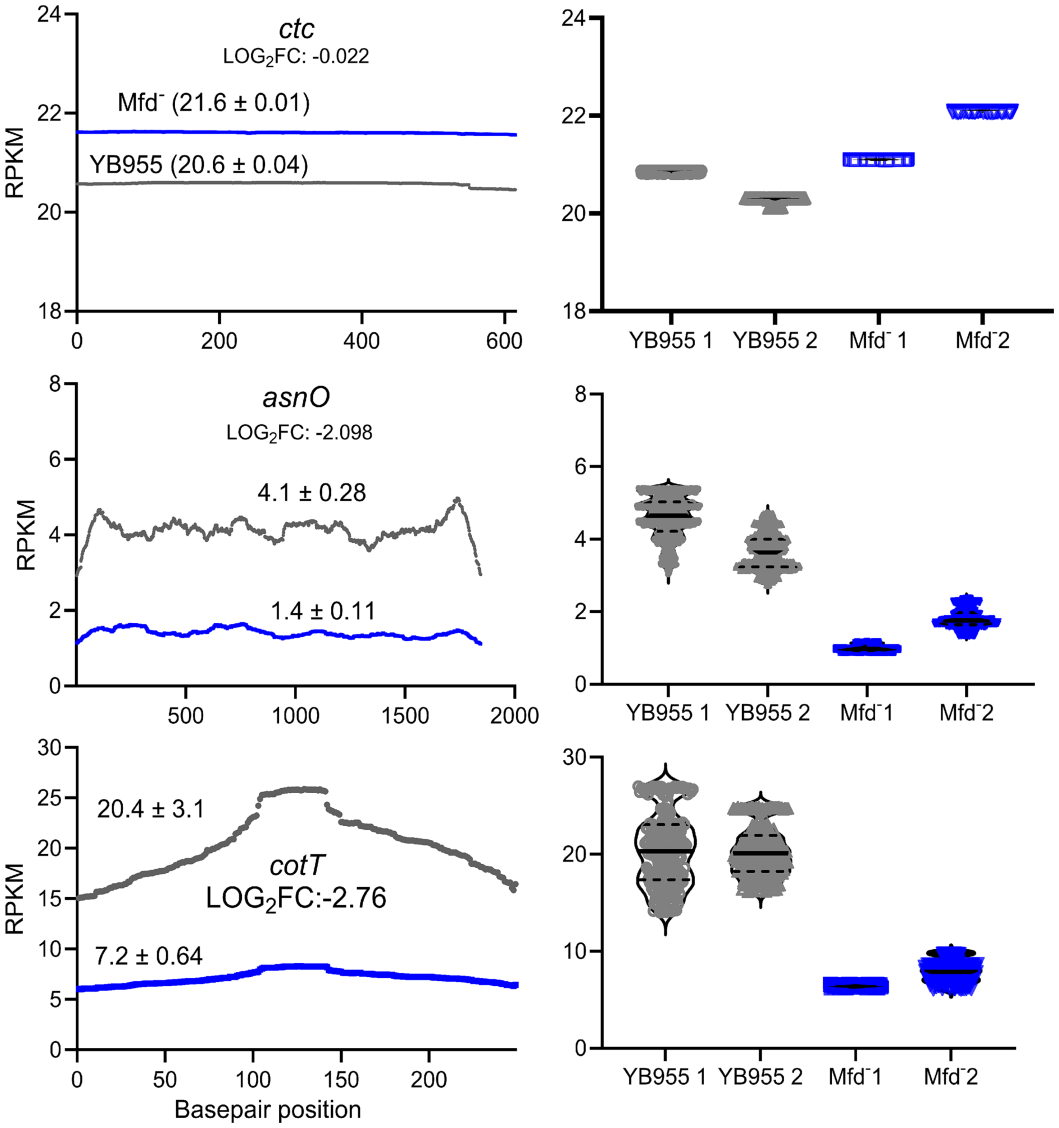
Effect of Mfd on the distribution of adjusted RNA-seq reads. (Left) Mean read distribution of two biological replicates as a function of gene base pair position. (Right) Violin plots showing the median and quartiles of RNA-seq reads in two biological replicates. Expression of the *ctc* gene is unaffected by Mfd and was used as a control. Expression of a*snO* and *cotT* is downregulated and shows decreased variance heterogeneity in the Mfd^−^ strain. Gray lines and dots represent the parental strain and blue lines and dots represent the Mfd^−^ strain.

Mfd functions as a modulator of RNAP during transcription elongation, interacts with paused RNAPs, and mediates transcript release or transcription reactivation (Deaconescu, 2021; Le et al., 2018). In a case in which RNAP pauses during transcription of coding sequences, Mfd may reactivate the paused RNAP into active transcription. In the context of RNA-seq read distribution, this would show as a large fluctuation in read coverage and the formation of complete transcripts. Then, we tested whether Mfd affects variability of transcription of coding sequences and the ability of RNAP to produce complete transcripts of genes affected during the process of sporulation. First, we reanalyzed read data from our previous report to measure differences in variability during transcription between the parent and Mfd^−^ cells (Martin et al., 2021). We hypothesized that Mfd deficiency alters fluctuations in read coverage of transcripts of genes affected by this factor—these included upregulated and downregulated genes as affected by six transcription activators controlling endospore formation (a total of 503 genes; Supplementary Table S1) (Elfmann et al., 2025). Specifically, we calculated the range in read coverage and the coefficient of variation (CV). Genes with an absolute difference greater than 2,000 or a greater than 11% CV across all three biological replicates were deemed to be notably different across their gene length (Figure 3). This procedure yielded 73 genes with a large fluctuation in read coverage across each gene between the two strains exclusive to either YB955 or the Mfd^−^ strain. Of those 73 genes, 31 were only observed in the parental strain (blue dots; Supplementary Table S2), and 42 were exclusive to the Mfd^−^ strain (red dots; Supplementary Table S3). Among the genes in which the range and CV were increased in YB955 but not in the Mfd^−^ strain, we identified genes activated during mid to late endospore formation, such as those encoding components of the cortex (*cot* genes) or factors involved in its assembly in both compartments. In addition, *spoV* genes (involved in transport of dipicolinic acid or remodeling of peptidoglycan layers in the developing spore) expressed in the forespore and *lytH*, encoding a peptidoglycan hydrolase expressed in the mother cell (Horsburgh et al., 2003), were affected by Mfd. Also, *asnO*, which encodes an asparagine synthetase (Yoshida et al., 1999) showed increased variability between the parent and the mutant (Martin et al., 2021). Based on these observations, and the microscopy and germination phenotypes, we focused on two sporulation genes transcriptionally activated by σ^E^ (controls gene expression in the mother cell), and whose expression was previously reported to be decreased as a function of Mfd. We analyzed expression of *asnO* and *cotT*. The *asnO* gene is associated with nutritionally supplementing the development of the spore, and its inactivation results in an asporogenous culture (Meeske et al., 2016). The *cotT* gene is, a structural component of the inner coat of the spore (Imamura et al., 2010; McKenney et al., 2013; McKenney et al., 2010). Inactivation of this gene does not affect the ability to germinate in the presence of L-alanine but results in spores with thinner coats (Bourne et al., 1991). We plotted the adjusted read coverage data of these genes at each base pair position and their distribution (Figure 4). The resulting profile revealed higher expression in the parent than in the Mfd^−^ mutant for both genes. Most importantly, we focused on the range and CV to ascertain whether transcription dynamics of coding sequences was affected by Mfd. The range was higher in the parent strain than in the mutant, and the quartile distribution suggested that the variances in read coverage in the parent and mutant were not homogeneous. These results suggest that transcription dynamics of coding sequences of *asnO* and *cotT* is affected by Mfd; perhaps transcription of these genes is highly variable (punctuated) because of Mfd-dependent transcriptional pausing, which would facilitate the formation of complete transcripts by the RNAP and produce optimal levels of mRNA of these genes. The same response was not observed in expression of *ctc*, a gene whose expression is unaffected by Mfd. The transcriptional response between the parent and the mutant for the *ctc* gene was almost identical, indicating that the effect of Mfd was specific to the *asnO* and *cotT* genes. We followed the read distribution analysis by examining the coding sequences of *asnO* and *cotT* for the potential to form secondary structures (non-B DNA and RNA structures) using in silico tools (see Materials and Methods). That analysis showed that *asnO* and *cotT* contain sequence motifs with the potential to form nucleic acid structures (ΔG of −5 or lower) that halt the elongating RNAP (Tornaletti et al., 2008) (see Supplementary Table S5). These structures were reported to associate with Mfd-dependent mutagenesis (Ermi et al., 2021), suggesting that Mfd is recruited to *asnO* and *cotT* during transcription.

**Figure 5:**
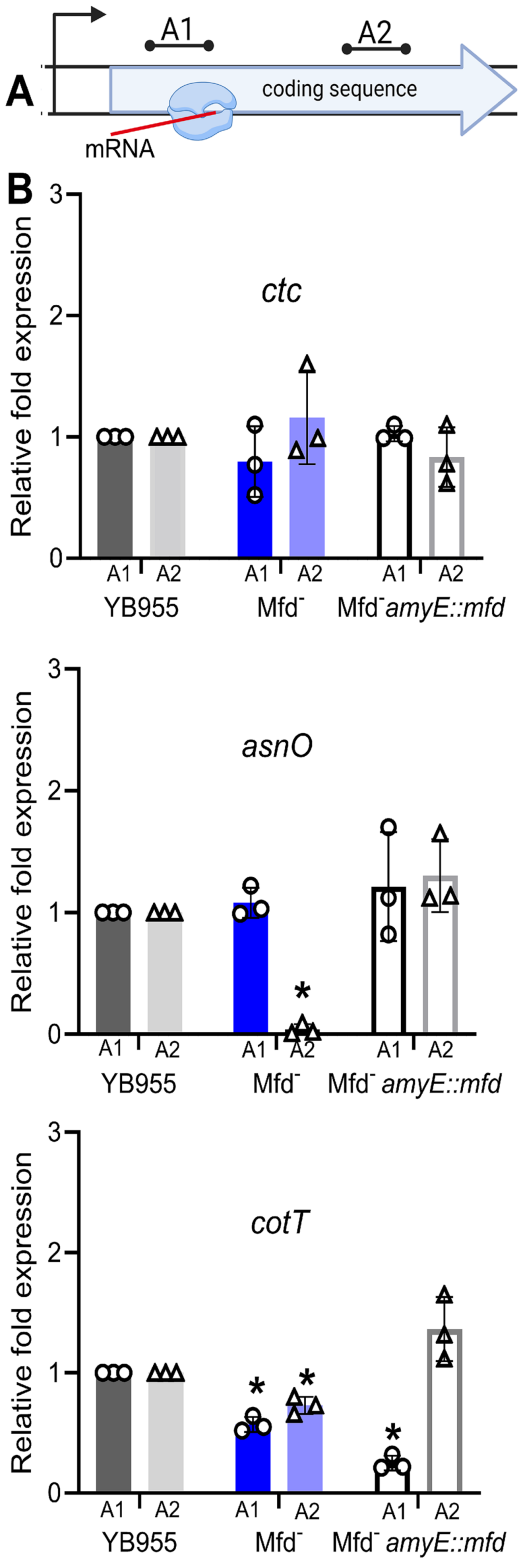
Effect of Mfd on expression of *ctc*, *asnO*, and *cotT* in strains differing in *mfd* status. **(A)** Diagram of RT-qPCR assay. **(B)** Relative transcript levels at two gene positions in *ctc*, *asnO*, and *cotT*. Restoration of Mfd results in wt levels of transcripts containing the 3′ end of the gene (complete transcripts). The *rnpB* gene was used to normalize gene expression. Relative gene expression was quantified using the 2^−ΔΔCT^ method. Bars show standard deviations from three biological replicates. Data was analyzed by one-way ANOVA. Means were tested by Tukey HSD. ^*^Indicates statistical significance at *p* ≤ 0.05.

To further test whether Mfd is a factor during the transcription of coding sequences and the formation of complete transcripts of the *asnO* and *cotT* genes, we selected two regions within these genes well beyond the promoter and transcriptional start site. We then performed RT-qPCR to quantify transcript abundance at two regions across these genes in various genetic backgrounds. Figure 5 shows the relative transcript levels for those regions. Deficiency of Mfd resulted in reduced levels of complete transcripts in both genes compared to the parent strain; abundance of the 3′ end of the transcript was significantly reduced (A2) compared to the parent strain and a control gene (*rnpB*; unaffected by Mfd). The transcript levels at the 3′ end of both genes were restored when a copy of *mfd* was integrated into the chromosome. Of note, transcript levels at the 5′ end of *cotT* were decreased in the *mfd*-restored strain (A1). Perhaps, overexpression of *mfd* indirectly affects degradation of mRNA at the 5′ end. In conjunction, these experiments indicate that Mfd alters the dynamics of transcription elongation of coding sequences of sporulation genes, and that its deficiency decreases the ability of RNAP to complete full transcripts of genes contributing to endospore formation.

## 4 Discussion

Previous studies have highlighted a role of Mfd in the development of spores, primarily by ensuring proper DNA repair (Ramirez-Guadiana et al., 2013; Ramírez-Guadiana et al., 2018; Valenzuela-García et al., 2018). Here, we report that Mfd optimizes gene expression important for sporogenesis, spore development, and maturation in cells unexposed to exogenous DNA damage. Results from TEM analyses showed that the absence of Mfd generated dormant spores with anomalous cortex features. Moreover, the defects in spore morphogenesis intersect with a decreased ability to complete transcripts in *asnO* and *cotT*; two *genes* activated during mid-late sporulation. Cortex formation is a critical step in *B. subtilis* sporulation, requiring tightly regulated expression and localization of peptidoglycan remodeling enzymes and scaffolding proteins (McKenney et al., 2013). Disruption of this process can lead to defective spores, particularly when the synthesis of its components, such as those encoded by the *cot* genes, is decreased. Moreover, our previous RNA-Seq dataset showed that several genes essential for cortex formation were significantly downregulated in the Mfd*^−^* strain (Martin et al., 2021). Notably, *spoVK*, a mother-cell-specific chaperone involved in spore maturation and activation of peptidoglycan synthesis showed decreased expression, and most importantly a decreased variation of transcription of its coding sequence in Mfd-deficient cells. These findings suggest that Mfd may promote proper cortex assembly by ensuring the completion of transcripts of key morphogenesis genes. Given that cortex development occurs in mid to late sporulation and involves precise spatial coordination between the mother cell and the forespore (Hilbert and Piggot, 2004), a decreased ability to complete transcripts of these genes could delay or disrupt morphogenesis.

The results shown here suggest that the defect seen in sporulation efficiency did not impact the ability of spores to return to vegetative growth during germination outgrowth (Figure 2). Interestingly, the DisA factor, a DNA damage scanning enzyme active during sporulation, suppresses the absence of Mfd for proper spore outgrowth (Valenzuela-García et al., 2018) when sporulating cells are exposed to DNA damage. Therefore, the lack of Mfd results in either a defect in spore maturation, resulting in a subpopulation of heat-sensitive spores, or a decreased percentage of cells initiating sporulation. Indeed, a previous report described a role for Mfd in coordinating a RecA-dependent checkpoint event during the onset of sporulation (Suárez et al., 2021). Future experiments testing heat activation of spores at different temperatures could discern if the lack of Mfd results in the development of spores that are more sensitive to heat than those produced by the parental strain.

The Mfd^−^ strain showed decreased levels of transcripts containing the 3′ ends of two genes activated by σ^E^ and encoding a component of the inner cortex, consistent with the TEM results showing reduced cortex thickness in this strain. Based on the results from the analyses of read distribution, variance heterogeneity, the potential for the formation of nucleic acid structures, and RT-qPCR, one possible mechanism explaining how Mfd influences sporogenesis is by processing transcription elongation events that delay or prevent the formation of complete transcripts of genes activated by the developmental transcription factors σ^E^ or σ^K^. Biochemical evidence has shown that Mfd contacts RNAPs halted by different events that include DNA lesions, road blocks consisting of DNA-protein complexes, elemental pauses, nucleotide deprivation, and nucleic acid secondary structures (Deaconescu et al., 2012; Haines et al., 2014; Kang et al., 2021; Landick, 2021). These events are resolved by reactivating transcription, which promotes the formation of complete transcripts, or prematurely terminating transcription and causing the release of an incomplete transcript. Upon directly encountering a halted RNAP and its DNA template, Mfd pushes it through repeated rounds of forward translocation. RNAP blocks formed by DNA-protein complexes or distorting DNA lesions are processed by terminating transcription prematurely. Alternatively, elemental transcriptional pauses, nucleotide deprivation, and DNA or RNA secondary structures, generated during transcription, can cause RNAP to backtrack and misalign the DNA template and the nascent mRNA (Kang et al., 2021; Le et al., 2018). Then, Mfd via its forward translocation activity facilitates realignment, reactivation of transcription by RNAP, and transcript completion. Given the highly-variable pattern of transcription of coding sequences, the presence of non-B DNA motifs previously shown to halt RNAP, and the RT-qPCR results, Mfd can promote expression of sporulation genes by reactivating backtracked RNAP elongation complexes into active transcription. This concept is supported by recent reports that show recruitment of Mfd to hard-to-transcribe regions (Ragheb et al., 2021).

Mfd is increasingly recognized not only as a transcription-coupled DNA repair factor, but also as a broader modulator of bacterial stress responses and cellular plasticity. In addition to its canonical role in resolving stalled transcription complexes, Mfd has been implicated in diverse cellular processes including stationary-phase mutagenesis, DNA recombination, and the evolution of antibiotic resistance (Ayora et al., 1996; Ragheb et al., 2019; Ross et al., 2006). These multifaceted functions suggest that Mfd acts as a developmental regulator, particularly under conditions of environmental stress (Pedraza-Reyes et al., 2024).

Although this study provides new insights into the role of Mfd in sporulation, several limitations should be considered. First, transmission electron microscopy (TEM) enables precise visualization and measurement of spore cortex thickness but captures only a static snapshot of mature spores and does not provide information on the dynamics or timing of cortex assembly. Additionally, TEM analyses may not fully capture population-level variability in spore morphology. Sample preparation steps, including fixation and dehydration, could also introduce minor artifacts that affect structural measurements, although standardized protocols were used to minimize these effects. Moreover, while cortex thickness serves as a useful morphological indicator of sporulation efficiency, we did not directly assess the functional consequences of this structural change, such as spore resistance to heat, desiccation, or chemical stress. Finally, our study focused on a subset of sporulation genes and phenotypes, and it remains unclear whether Mfd influences other aspects of spore maturation such as core dehydration or dipicolinic acid accumulation. Future studies incorporating broader structural and functional assays will be important to fully define the role of Mfd in spore development. Further experiments could test resistance phenotypes such as heat, UV, or lysozyme sensitivity to see if cortex alteration correlates with decreased stress resistance. It remains to be seen whether Mfd plays a similar morphogenetic role in other spore-forming species, such as *Clostridioides difficile*, which could inform new strategies to inhibit spore formation in pathogens.

## Data Availability

All generated data and code are publicly available via GitHub (https://github.com/Robleto-Lab/mfd-sporulation-dashboard/). These sporulation-affected genes can be accessed via a user interface (https://robleto-lab.github.io/mfd-sporulation-dashboard/).
