# Supplementary figures and images for: Mfd deficiency decreases the abundance of complete transcripts of sporulation genes and alters sporogenesis and the structure of dormant Bacillus subtilis spores
Source: Front Microbiol. 2025 Oct 17;16:1680580. doi: 10.3389/fmicb.2025.1680580 (PMC12575378; doi:10.3389/fmicb.2025.1680580)

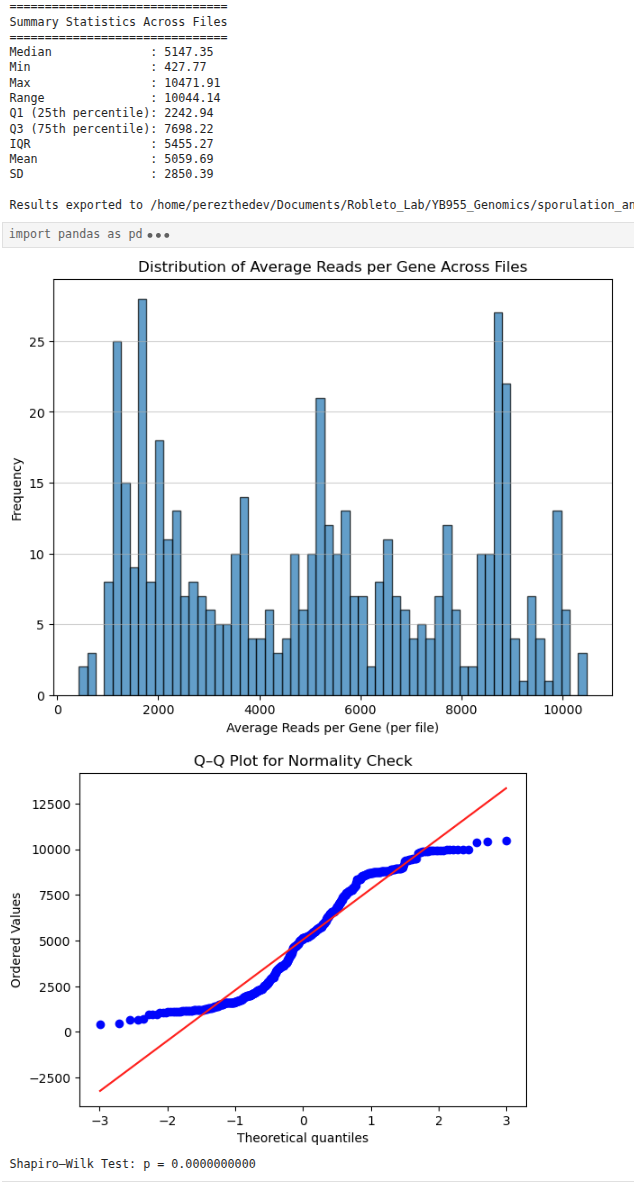

Supplement: Supplementary file 1 [file Image_1.PNG]
