# Supplementary material for: Mfd deficiency decreases the abundance of complete transcripts of sporulation genes and alters sporogenesis and the structure of dormant Bacillus subtilis spores
Source: Front Microbiol. 2025 Oct 17;16:1680580. doi: 10.3389/fmicb.2025.1680580 (PMC12575378; doi:10.3389/fmicb.2025.1680580)
Supplement: Supplementary file 6 [file Table_5.DOCX]

| Table 6. DNA motifs and their likelihood of forming non-B DNA structures. | | | | | |
| --- | --- | --- | --- | --- | --- |
| Gene | Sequence | Motif | Position | RNA ΔG  kcal/mol | DNA ΔG  kcal/mol |
| *asnO* | GGCGGGTACCCGTGGTTCCATACGG | G-rich | 1156-1181 | -5.40 | -3.15 |
| *asnO* | GGACGCGGTGACGCGGCAGCTTGTGTCGG | G-rich | 741-770 | -10.26 | -5.92 |
| *asnO* | GGTCCCGTGGTTCGGGCAGCTCATGAAGG | G-rch | 1740-1769 | -7.35 | -4.54 |
| *asnO* | GGCTTTCCATGGATGAGATCAACGGAGG | G-rich | 1195-123 | -5.46 | -1.77 |
| *cotT* | GGTTACGGCGGAGGTTATGG | G-rich | 199-219 | -6.46 | -1.16 |
| *cotT* | GGATATGGGGGAGGTTACGG | G-rich | 223-243 | -6.71 | -2.02 |
| *ctc* | NONE |  |  |  |  |
